# Immediate Effects of Magnetic Stimulation on Dentate Gyrus Glutamatergic and GABAergic Neuron Excitability

**DOI:** 10.3390/brainsci16070673

**Published:** 2026-06-26

**Authors:** Zihao Ren, Boya Lu, Haoyu Qiu, Zixuan Wang, Tianjiu Wang, Jiale Kang, Teng Zou, Haijun Zhu, Chong Ding

**Affiliations:** 1 College of Health Sciences & Biomedical Engineering, Hebei University of Technology, Tianjin 300130, China; 202322902007@stu.hebut.edu.cn (Z.R.);; 2 Key Laboratory of Bioelectromagnetics and Neural Engineering of Hebei Province, Hebei University of Technology, Tianjin 300130, China; 3 Key Laboratory of Digital Medical Engineering of Hebei Province, College of Electronic & Information Engineering, Hebei University, Baoding 071002, Chinazhuhj@hbu.edu.cn (H.Z.)

**Keywords:** magnetic stimulation, GABAergic neuron, glutamatergic neuron, action potential, Hodgkin–Huxley model

## Abstract

**Highlights:**

**What are the main findings?**

- High-frequency magnetic stimulation immediately enhances the excitability of both glutamatergic and GABAergic neurons in the hippocampal dentate gyrus, with GABAergic neurons exhibiting greater sensitivity to changes in stimulation parameters.
- Computational simulations using an improved Hodgkin–Huxley model reproduced both the weak effects under 1 Hz stimulation and the excitability-enhancing effects under high-frequency stimulation, suggesting that frequency-dependent changes in sodium and potassium channel gating kinetics may contribute to the observed responses.

**What are the implications of the main findings?**

- This provides crucial insights into how magnetic stimulation modulates the imbalance between neural excitation and inhibition associated with hippocampus-related cognitive disorders.
- Elucidating the intrinsic biophysical mechanisms at the level of individual ion channels can provide a theoretical foundation for optimizing therapeutic stimulation parameters in clinical applications.

**Abstract:**

**Background/Objectives**: To investigate the immediate regulatory effects of magnetic stimulation with different parameters on the excitability of glutamatergic neurons and GABAergic neurons in the mouse hippocampal dentate gyrus (DG), and to analyze the underlying mechanisms using the Hodgkin–Huxley (HH) model. **Methods**: Whole-cell patch-clamp recordings were performed on acute brain slices to measure changes in resting membrane potential (RMP), the number of action potentials (APs) evoked by 500-ms long-duration stimulation, as well as AP threshold, peak, half-width, maximum rising slope, and maximum falling slope under magnetic stimulation at various frequencies (1, 10, 20 Hz) and intensities (50, 75 mT). An improved HH model was established based on experimental data to analyze the dynamic changes in gating variables under magnetic stimulation. **Results**: High-frequency magnetic stimulation (10–20 Hz) significantly increased the number of APs in both neuron types. In glutamatergic neurons, the number of APs increased from 10.12 ± 0.52 in the control group to 15.62 ± 0.84 in the 20 Hz-75 mT group; in GABAergic neurons, it increased from 7.88 ± 0.40 to 12.62 ± 0.53. Magnetic stimulation also depolarized RMP and significantly altered multiple AP waveform parameters in both neuron types. Glutamatergic neurons showed a more distinct frequency dependence, whereas GABAergic neurons were more sensitive to changes in both frequency and intensity in terms of RMP and multiple waveform parameters. Simulation results showed that the 1 Hz conditions produced negligible changes in AP firing, gating-variable dynamics, and steady-state ion-channel parameters compared with the Control condition. In contrast, high-frequency stimulation enhanced the dynamic changes of sodium and potassium channel gating variables and altered their voltage-dependent steady-state properties. Specifically, sodium channel activation shifted toward more negative potentials, whereas sodium channel inactivation and potassium channel activation shifted toward more depolarized potentials. **Conclusions**: Under the experimental conditions of this study, magnetic stimulation immediately enhanced the excitability of glutamatergic and GABAergic neurons in the hippocampal dentate gyrus of male mice in a frequency-dependent manner. The modified HH model reproduced both the weak effects under low-frequency stimulation and the enhanced excitability under high-frequency stimulation, suggesting that these immediate effects may be related to frequency-dependent changes in the gating kinetics and voltage-dependent properties of sodium and potassium channels.

## 1. Introduction

With the rapid development of neuroscience and neuroengineering, modulating nervous system activity using exogenous physical means has become an important direction in basic research and clinical applications [1]. Magnetic stimulation exhibits unique value in neuromodulation research due to its advantages, which include non-contact application, strong penetration capability, flexibly adjustable stimulation parameters, and minimal tissue damage [2]. As a typical application of magnetic stimulation, repetitive transcranial magnetic stimulation (rTMS) can induce relatively long-lasting neuromodulatory effects by continuously applying magnetic pulses at specific frequencies and rhythms [3].

Despite the widespread application of rTMS, its underlying mechanisms are not fully elucidated. Existing studies largely focus on post-stimulation changes in brain region function, network reorganization, and long-term plasticity, while the understanding of the cellular electrophysiological mechanisms during the initial stages of magnetic stimulation remains insufficient [4]. Research indicates that alterations in neuronal excitability may be one of the key mechanisms underlying the therapeutic effects of rTMS [5,6]. rTMS can directly or indirectly alter the membrane potential of target neurons [7] and reshape neuronal connections; prolonged stimulation can produce cumulative effects leading to long-term neural plasticity changes, and these effects likely originate from the immediate modulation of neuronal excitability by magnetic stimulation [8].

The hippocampus is a crucial brain region for learning and memory, within which the dentate gyrus (DG) plays a pivotal role in cognitive function and is susceptible to modulation by magnetic stimulation. The DG region is rich in excitatory glutamatergic neurons and inhibitory GABAergic neurons. The excitation/inhibition imbalance caused by the abnormal functioning of these two types of neurons is a major cause of various diseases [9]. Understanding the immediate responses of DG neurons to magnetic stimulation may provide experimental evidence for optimizing magnetic stimulation paradigms and may contribute to the future development of neuromodulation strategies for hippocampus-related disorders [10,11]. Different types of neurons vary in morphological structure [12], membrane electrical properties, ion channel expression, and firing patterns; therefore, their responses and kinetic processes under magnetic stimulation may also differ [13].

To deeply investigate neuromodulatory mechanisms, previous studies have utilized optogenetic techniques, which can precisely control the activity of specific neuronal populations with extremely high spatiotemporal resolution. By specifically expressing light-sensitive ion channels or inhibitory proteins in mouse models, specific wavelengths of light are used to precisely activate or inhibit the firing of glutamatergic or GABAergic neurons [14,15]. Microelectrode arrays and patch-clamp electrophysiology techniques can record changes in firing activity and synaptic transmission of specific neurons in acute brain slices following rTMS [16]. The Hodgkin–Huxley (HH) model can accurately replicate the generation process of neuronal action potentials (APs) by precisely simulating the dynamic characteristics of voltage-gated sodium, potassium, and leak channels. Its simulation results can well reproduce experimental data from in vitro electrophysiology, holding significant biophysical relevance [17,18]. Yang et al. [19] theoretically predicted changes in ion channel kinetics and firing patterns under magnetic field stimulation by improving the HH neuron model under a sinusoidal magnetic field. However, although existing computational models attempt to simulate the effects of magnetic fields on neuronal membrane potential, they have not been closely integrated with electrophysiological experiments to systematically explain experimental phenomena and reveal deep ion channel kinetic mechanisms. Currently, there is still a lack of sufficient mechanistic understanding regarding the cellular-level modulatory patterns of the immediate effects of magnetic stimulation and their parameter dependence. Low-frequency magnetic stimulation is generally considered to inhibit cortical excitability, while high-frequency stimulation is thought to enhance it, but this rule still needs verification in the immediate modulation of different neuronal types.

Therefore, this study focuses on the following questions: whether magnetic stimulation can immediately enhance neuronal excitability; whether this modulation is frequency- and intensity-dependent; and whether there are differences in the responses of neurons with different functional types to magnetic stimulation. Based on this, the present study constructed a comprehensive research framework primarily based on immediate in vitro electrophysiological experiments, supplemented by computational simulations for explanation. Combining whole-cell patch-clamp techniques, we recorded changes in the resting membrane potential (RMP), the number of APs fired, and waveform parameters of glutamatergic and GABAergic neurons following magnetic stimulation at different frequencies and intensities. On this basis, an improved HH model was utilized to provide a computational interpretation and a potential mechanistic explanation of the experimental findings, aiming to elucidate the modulatory rules and potential underlying mechanisms of magnetic stimulation on the excitability of these two types of neurons, thereby providing experimental evidence for understanding the immediate cellular responses to magnetic stimulation and informing future optimization of stimulation parameters.

## 2. Materials and Methods

### 2.1. Animals and Groups

All animal experiments were approved by Hebei University of Technology and complied with the guidelines for the care and use of laboratory animals (Ethics Approval No. HEBUTaCUC2025025). Male Kunming mice, 8 weeks old and weighing (32 ± 3) g, were used in this experiment. The mice were purchased from Henan Skbion Biotechnology Co., Ltd., Zhengzhou, China. The animals were housed in a fully automated environmental control system with the room temperature maintained at 25 °C and a 12-h light/12-h dark cycle. They had ad libitum access to sterilized pellet feed and purified water. Prior to the formal experiments, the mice were acclimatized for one week.

A total of 30 mice were used in this study, of which 13 were used for virus injections related to glutamatergic neurons, 13 for virus injections related to GABAergic neurons, and the remaining 4 for control-related experiments. The mice first underwent viral injections for neuronal type identification. In the experiments, the recombinant adeno-associated virus rAAV-CaMKIIα-GCaMP6m-WPRE-hGH polyA (Wuhan Brain VTA Co., Ltd., Wuhan, China) was used as the viral tool to identify candidate glutamatergic neurons, and rAAV-VGAT1-GCaMP6m-WPRE-hGH polyA (Wuhan Brain VTA Co., Ltd., China) was used to identify candidate GABAergic neurons. Following anesthesia, the mice underwent stereotaxic injection. The target area for viral injection was the dentate gyrus (DG) of the hippocampus. The injection coordinates were 1.2 mm to the right of the midline, 1.7 mm posterior to the bregma, and at a depth of 2.0 mm, with 200 nL of the virus injected per side. After surgery, the mice were returned to their cages and housed for another 3 weeks to ensure adequate viral expression.

Brain slices were prepared upon the completion of viral expression. Approximately 8 brain slices could be obtained from each mouse, yielding a total of about 240 brain slice samples. The brain slices were divided into the Control group, 1 Hz-50 mT group, 10 Hz-50 mT group, 20 Hz-50 mT group, 1 Hz-75 mT group, 10 Hz-75 mT group, and 20 Hz-75 mT group. Each brain slice received magnetic stimulation at most once and underwent a single whole-cell patch-clamp recording. During the experiment, combined with real-time observation under a fluorescence microscope, the target cells expressing GCaMP6m exhibited green fluorescence signals under blue light excitation. Based on this, candidate target neurons were localized, and electrophysiological recordings were performed. The viral targeting strategy employed here is a well-established method with robust and extensive supporting literature. The high cell-type specificity of these short promoters in hippocampal regions has been repeatedly validated in numerous top-tier international studies [20,21,22,23,24].

Approximately eight coronal hippocampal slices containing the dentate gyrus were obtained from each mouse. These slices were allocated across the different experimental groups whenever possible. Prior to recording, slices were evaluated for structural integrity, preservation of the dentate gyrus, adequate viral fluorescence expression, and suitability for stable whole-cell patch-clamp recording. Slices failing to meet these criteria or yielding unstable recordings were excluded from analysis. Because losses during slice preparation and electrophysiological recording are unavoidable, additional animals were prepared to ensure sufficient biological replicates for each experimental group. To avoid pseudoreplication, no more than one brain slice from the same mouse contributed to any individual experimental group.

In this study, the sample size for each experimental group was *n* = 8, where *n* represents eight independent electrophysiological recordings obtained from eight different brain slices. Each data point corresponds to one successfully recorded neuron from one brain slice. Each brain slice received magnetic stimulation only once and was used for only one electrophysiological recording. Within each experimental group, all recordings were obtained from different brain slices originating from different experimental mice.

To ensure that stimulation frequency was the primary variable while minimizing differences in the total stimulation dose, all magnetic stimulation protocols were standardized to 200 pulses. Specifically, the 1 Hz protocol consisted of 200 consecutive pulses delivered continuously over approximately 200 s (3 min 20 s). The 10 Hz protocol consisted of 10 stimulation trains, each containing 20 pulses delivered over 2 s, with an inter-train interval of 5 s. The 20 Hz protocol also consisted of 10 stimulation trains, each containing 20 pulses delivered over 1 s, separated by 5 s inter-train intervals. Whole-cell patch-clamp recordings were initiated immediately after completion of the magnetic stimulation protocol. This design ensured that all stimulation groups received the same total number of pulses, allowing the effects of stimulation frequency and magnetic field intensity on neuronal electrophysiological properties to be compared independently.

### 2.2. Magneto-Electrophysiological Coupling System

**Figure 1:**
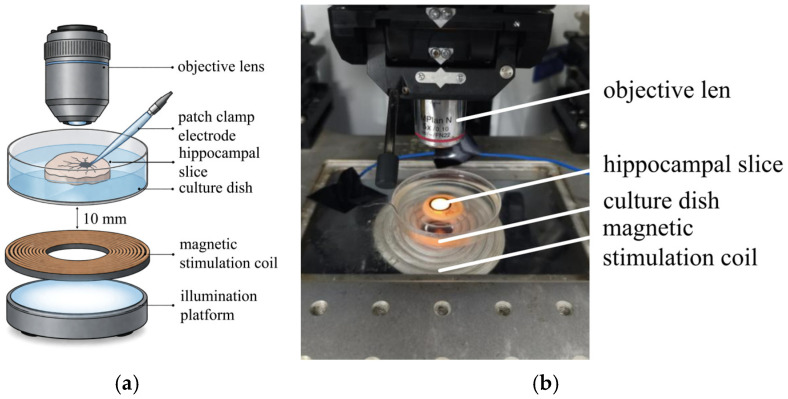
Schematic and actual view of the integrated magnetic stimulation and electrophysiology system. (**a**) Schematic diagram. (**b**) Actual photo.

To facilitate combined magnetic stimulation and whole-cell patch-clamp recordings in acute brain slices, an integrated magnetic stimulation and electrophysiology system was employed for stimulation. This system consists of a customized miniature circular coil and a patch-clamp platform. It allows for the application of magnetic stimulation to the target regions of the brain slice without interfering with microscopic observation or electrode manipulation. Figure 1 illustrates the schematic and photographic representation of the integrated magnetic stimulation and electrophysiology system.

#### 2.2.1. Magnetic Stimulation Coil Design and Stimulation Implementation

The coil used in the experiment was a miniaturized single-layer axisymmetric circular coil adapted for the patch-clamp platform, utilizing a 6-turn winding structure. The inner radius of the coil was 12.5 mm, the outer radius was 40 mm, and the radial width was 27.5 mm. The coil was positioned above the bottom light source of the patch-clamp setup. During the procedure, the center of the hollow coil was kept coaxial with the bottom light source, allowing the brain slice to be positioned approximately 10 mm directly above the central axis of the coil. The coil plane was maintained parallel to the culture dish to ensure the target area remained within the primary stimulation range. The design of the miniaturized circular coil was based on electromagnetic induction principles and previous coil-design strategies for magnetic stimulation, in which coil geometry, winding distribution, stimulation distance, and induced electric-field distribution are key determinants of stimulation focality and intensity [25,26,27].

The relevant design principles and the derivation of geometric parameters have been systematically demonstrated in our research group’s previous work on the construction of the integrated magnetic stimulation and electrophysiology system.

**Figure 2:**
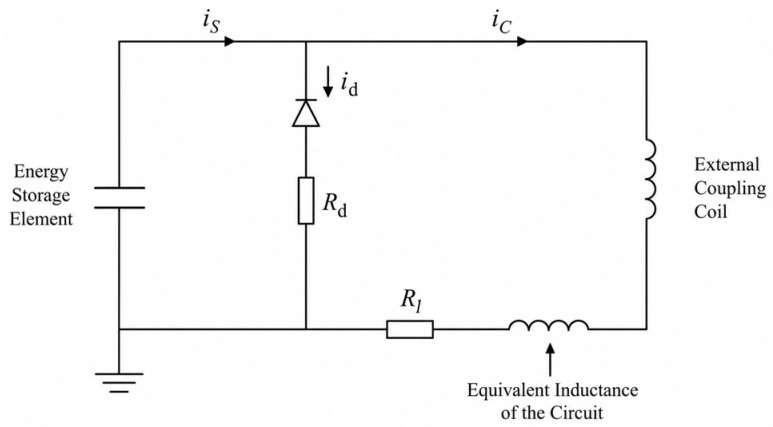
RLC pulsed magnetic field generating circuit.

The coil was driven by an RLC pulse discharge circuit, and various magnetic stimulation intensities were achieved by adjusting the initial charging voltage of the capacitor. Under an initial charging voltage of 100 V, the peak discharge current of the RLC circuit was approximately 1013.3 A, which closely approached the design target of 1000 A. The rising edge of the magnetic field in the coil’s target area was approximately 50 μs, and the falling edge was approximately 250 μs; thus, the system was capable of generating short-duration pulsed magnetic stimulation. The RLC pulsed magnetic field generator circuit is shown in Figure 2. The symbols are defined as follows: *i_S_*, discharge current; *i_C_*, coil-branch current; *i_d_*, diode-branch current; *R_d_*, damping/protection resistor; and *R_l_*, equivalent circuit resistance.

The in vitro brain slice experiments utilized two magnetic field intensity levels, 50 mT and 75 mT, with stimulation frequencies set at 1 Hz, 10 Hz, and 20 Hz. According to the simulation results, under the 50 V condition, a peak magnetic field of approximately 53 mT and a peak induced electric field of approximately 25 V/m could be generated at the target area about 10 mm above the central axis of the coil. Under the 70 V condition, a peak magnetic field of approximately 73 mT and a peak induced electric field of approximately 37 V/m could be generated. The output magnetic field and induced electric field of the coil varied with the initial charging voltage, fulfilling the experimental requirements for the 50 mT and 75 mT magnitude stimulation conditions applied in this study. In the experiments, the Control group received sham stimulation, in which the coil was kept at approximately 80–100 mm from the brain slice, though non-specific interferences, such as the acoustic noise generated during the operation of the device, were retained.

#### 2.2.2. Simulation and Verification of Coil Electromagnetic Fields and Thermal Effects

A three-dimensional finite element model of the coil was established using COMSOL Multiphysics 6.2. With the RLC transient discharge current acting as the excitation source, the magnetic field, electric field, and time-domain variation characteristics in the target area of the coil were simulated and analyzed. A stable pulsed magnetic field could be formed approximately 10 mm above the central axis of the coil. The intensities of the magnetic field and the induced electric field varied correspondingly with the initial charging voltage, enabling continuous adjustment under different intensity stimulation conditions. Influenced by the axisymmetric circular coil structure, the vector sum of the induced electric field on the central axis was zero, and the peak electric field was primarily distributed in the radial peripheral region of the coil. This distribution was capable of covering the target stimulation area of the mouse brain slices. The target stimulation area is defined as the region of an acute brain slice containing the DG that lies within the illuminated field of view. In this experimental setup, the brain slice has a radius of approximately 0.3 mm, which corresponds to a cross-sectional area of approximately 0.09 square mm^2^.

A coupled electromagnetic field-solid heat transfer-fluid flow model was employed to evaluate the thermal effects of the homotypic coil system. Under typical working conditions, the temperature rise in the coil following heat dissipation optimization was significantly reduced: under the conditions of 10 Hz, 100 V, and 4 s, the maximum temperature of the coil was controlled within 22.5 °C, with a relative environmental temperature rise of ≤0.5 °C; during continuous operation for 10 s, the maximum temperature could still be controlled within 26 °C. This system can, to a certain extent, mitigate the risk of thermal interference during continuous stimulation, providing relatively stable hardware support for in vitro brain slice patch-clamp experiments.

In summary, the integrated magnetic stimulation and electrophysiology system utilized in this study possesses a clear methodological basis in terms of geometric structure, stimulation waveform, electromagnetic field output, and temperature rise control, thereby satisfying the fundamental requirements for combined in vitro brain slice magnetic stimulation and whole-cell patch-clamp experiments.

### 2.3. Patch-Clamp Electrophysiology Experiments

#### 2.3.1. Preparation of Brain Slices

Mice were deeply anesthetized with 5% isoflurane and rapidly decapitated. The brains were extracted, placed into an oxygenated cutting solution, and chilled. Subsequently, the brain tissue was sectioned into 300-μm-thick brain slices using a VT1200S vibratome (Leica Biosystems, Nußloch, Germany). The brain slices were then incubated in an incubation chamber containing standard artificial cerebrospinal fluid (ACSF) continuously bubbled with a gas mixture of 95% O_2_ and 5% CO_2_, with the temperature maintained at 30 °C [28]. The formulations of the ACSF, internal solution of AP, and cutting solution used during the patch-clamp electrophysiology experiments are presented in Table 1.

#### 2.3.2. Electrophysiological Procedures

Following a one-hour incubation period, the brain slices were carefully transferred into a recording dish using a pipette, and a nylon mesh was gently placed over the slices to prevent movement. The dish was then placed on the stage of the patch-clamp system. Under a fluorescence microscope, glutamatergic and GABAergic neurons in the DG region were identified. The internal solution was loaded into the pulled glass pipettes, and the initial pipette resistance was verified to be within 5–10 MΩ using the PatchMaster software (version 2x73.2). The pipette tip was advanced to contact the target cell membrane with slight pressure. Positive pressure was subsequently released until a gigaseal (resistance > 1 GΩ) was formed. Fast capacitance compensation was then performed. Negative pressure was applied to rupture the membrane and achieve the whole-cell configuration. Once the access resistance stabilized at approximately 200–300 MΩ, slow capacitance compensation was applied to establish a stable whole-cell recording.

Baseline (pre-stimulation) signals were recorded before the application of magnetic stimulation. Immediately following stimulation, the RMP and AP-related parameters were recorded. We analyzed the neuronal RMP, the number of APs evoked during 500-ms long-duration current pulses, and individual AP parameters, including threshold, peak, half-width, time-to-peak, and the maximum rising and falling slopes. All electrophysiological data were acquired and recorded using HEKA PatchMaster software (version 2x73.2). The firing frequency was analyzed based on the AP counts evoked by the long-duration current pulses prior to magnetic stimulation, which served as the basis for further refining the HH neuron models for both types of neurons.

### 2.4. Electrophysiological Simulation Methods

#### 2.4.1. The HH Model Under the Influence of a Magnetic Field

To interpret the experimentally observed changes in neuronal excitability under magnetic stimulation, we adopted a modified Hodgkin–Huxley model based on the computational framework proposed by Yang et al. [19]. In that study, magnetic-field-dependent correction terms were introduced into the ionic current equations by extending the classical HH formulation. In the present study, this previously published formulation was employed as a phenomenological model to reproduce the experimentally observed electrophysiological responses rather than as a first-principles electromagnetic description of magnetic stimulation. The modified HH neuron model is formulated as follows:

(1) $$C_{m}\frac{dV}{dt}=I_{\mathrm{ext}}-I_{\mathrm{Na}}-I_{K}-I_{L}+\Delta I_{\mathrm{Na}}+\Delta I_{K}+\Delta I_{L}$$

(2) $$\frac{dm}{dt}=\alpha_{m}\left(V\right)\left(1-m\right)-\beta_{m}\left(V\right)m$$

(3) $$\frac{dh}{dt}=\alpha_{h}\left(V\right)\left(1-h\right)-\beta_{h}\left(V\right)h$$

(4) $$\frac{dn}{dt}=\alpha_{n}\left(V\right)\left(1-n\right)-\beta_{n}\left(V\right)n$$

(5) $$I_{\mathrm{Na}}=G_{\mathrm{Na}}m^{3}h\left(V-V_{\mathrm{Na}}\right)$$

(6) $$I_{K}=G_{K}n^{4}\left(V-V_{K}\right)$$

(7) $$I_{L}=G_{L}\left(V-V_{L}\right)$$

Equation (1) describes the variation in membrane potential *V* over time, where *C*_m_ is the lipid bilayer capacitance and *I*_ext_ is the externally applied current, representing external neural stimulation delivered via a current source. In Equations (2)–(4), *m* and *h* are the gating variables for voltage-gated sodium channels, representing the activation and inactivation of these channels, respectively. *n* is the gating variable for voltage-gated potassium channels, representing the activation of the potassium channel current. *G*_Na_, *G*_K_, and *G*_L_ denote the maximum conductances of the sodium, potassium, and leak ion currents, respectively. *V*_Na_, *V*_K_, and *V*_L_ represent the reversal potentials of the sodium, potassium, and leak channels, respectively. *α* and *β* are rate functions that depend solely on the membrane potential. *α_m_* and *β_m_* are the opening and closing rates of the Na+ channel activation gate; *α_h_* and *β_h_* are the opening and closing rates of the Na+ channel inactivation gate; and *α_n_* and *β_n_* are the opening and closing rates of the K+ channel activation gate. The terms $\Delta I_{\mathrm{Na}}$, $\Delta I_{K}$, and $\Delta I_{L}$ in Equation (1) represent the variations in sodium, potassium, and leak ion currents under the influence of a magnetic field, with their formulas given as follows:

(8) $$\Delta I_{\mathrm{Na}}=\mu_{\mathrm{Na}}I_{\mathrm{Na}}B_{\mathrm{ext}}$$

(9) $$\Delta I_{K}=\mu_{K}I_{K}B_{\mathrm{ext}}$$

(10) $$\Delta I_{L}=-\mu_{L}I_{L}B_{\mathrm{ext}}$$

where *B*_ext_ represents the external magnetic field. The pulse width of magnetic stimulation is a critical parameter that influences its physiological and therapeutic effects [29]. Pulse width defines the duration of the stimulation pulse, typically expressed in microseconds (µs) [30]. The pulse widths of magnetic stimulation at various frequencies range from approximately 80 to 500 µs [31]. For computational convenience, incorporating actual pulse width data, the average magnetic field pulse width was set to 200 µs with a high-frequency half-wave sine waveform (with an internal frequency of 5000 Hz, corresponding to the pulse width calculation). During implementation, we used the magnetic field intensity as the pulse amplitude and the pulse frequency as the stimulation application frequency, modulating the ion currents via magnetic induction coefficients (*μ*_Na_, *μ*_K_, and *μ*_L_). Here, *μ*_Na_, *μ*_K_, and *μ*_L_ represent the mobilities of sodium, potassium, and leak ions, respectively, where *μ*_Na_ = 3 m^2^/(V·s), *μ*_K_ = 3.8 m^2^/(V·s), and *μ*_L_ = 1 m^2^/(V·s) [19].

#### 2.4.2. Parameter Fitting Based on AP Characteristics

By adjusting the rate functions, membrane potential, as well as the conductances and reversal potentials of the sodium, potassium, and leak channels, the HH model can represent the AP firing behaviors of different types of neurons [32]. Based on existing literature and combined with the RMP and AP-related parameters extracted from patch-clamp recordings, this study further fitted and modified the model to obtain a neuron model that better aligns with the characteristics of our experimental data. The initial parameters of the model referred to the classical HH framework and the model parameters of glutamatergic and GABAergic neurons in the hippocampus. The reversal potentials were set according to physiological ranges, while the conductance parameters and the kinetic parameters of the gating variables were used to adjust the AP waveforms and firing behaviors.

By comparing the model output with the experimental measurements, the parameters were iteratively fitted so that the model’s AP firing characteristics approached the experimental observations as closely as possible. Due to the limited detailed electrophysiological model parameters available for granule cells in the hippocampal dentate gyrus, the parameters for glutamatergic neurons referred to the HH model of pyramidal neurons in the hippocampal CA1 region, and were further fitted in combination with our experimental results [17]. For GABAergic neurons, parameters were further adjusted based on existing literature models to match the AP characteristics obtained from our patch-clamp recordings [32]. Through the aforementioned process, parameterized models for the two types of neurons were ultimately obtained. The parameters regarding membrane potentials, as well as the conductances and reversal potentials of the sodium, potassium, and leak channels for both types of neurons, are presented in Table 2. The rate functions for glutamatergic neurons are defined as follows:

(11) $$\alpha_{m}\left(V\right)=\frac{0.1\left(38+V\right)}{1-\exp\left(-\frac{38\;+\;V}{10}\right)}\;\mathrm{and}\;\beta_{m}\left(V\right)=4.0\exp\left(-\frac{63+V}{18}\right)$$

(12) $$\alpha_{n}\left(V\right)=\frac{0.01\left(V+34\right)}{1-\exp\left(-\frac{V\;+\;34}{10}\right)}\;\mathrm{and}\;\beta_{n}\left(V\right)=0.125\exp\left(-\frac{V+44}{80}\right)$$

(13) $$\alpha_{h}\left(V\right)=0.07\exp\left(-\frac{V+60}{18}\right)\;\mathrm{and}\;\beta_{h}\left(V\right)=\frac{1}{1+\exp\left(-\frac{V\;+\;30}{10}\right)}$$

The membrane potential-dependent rate functions for GABAergic neurons are defined as follows:

(14) $$\alpha_{m}\left(V\right)=\frac{-0.1\left(38+V\right)}{\exp\left(-\frac{38\;+\;V}{10}\right)-1}\;\mathrm{and}\;\beta_{m}\left(V\right)=4\exp\left(-\frac{58+V}{14}\right)$$

(15) $$\alpha_{n}\left(V\right)=\frac{-0.01\left(30+V\right)}{\exp\left(-\frac{30\;+\;V}{10}\right)-1}\;\mathrm{and}\;\beta_{n}\left(V\right)=0.125\exp\left(-\frac{40+V}{30}\right)$$

(16) $$\alpha_{h}\left(V\right)=0.07\exp\left(-\frac{V+55}{10}\right)\;\mathrm{and}\;\beta_{h}\left(V\right)=\frac{1}{\exp\left(-\frac{V\;+\;28}{10}+1\right)}$$

**Table 2:** Neuron model parameters.

Parameters	Description	Excitatory Neuron Parameter Values	Inhibitory Neuron Parameter Values
*E*_L_	Leakage Channel Reversal Potential	−70 mV	−68 mV
*E*_Na_	Sodium Ion Channel Reversal Potential	50 mV	48 mV
*E*_K_	Potassium Ion Channel Reversal Potential	−90 mV	−87 mV
*G*_L_	Leakage channel conductance	0.18 mS·cm^−2^	0.18 mS·cm^−2^
*G*_Na_	Sodium Ion Channel Conductance	42 mS·cm^−2^	42 mS·cm^−2^
*G*_K_	Potassium Ion Channel Conductance	9 mS·cm^−2^	7.1 mS·cm^−2^
*C*_m_	Cell membrane capacitance	1 μF·cm^−2^	1 μF·cm^−2^

#### 2.4.3. Simulation Implementation Steps

Based on the actual firing frequencies of the two types of neurons in the absence of a magnetic field in the experiment, the external input current was calibrated in the improved HH model to match the observed firing characteristics. Numerical simulations were performed using MATLAB R2023b to solve the ordinary differential equations of the HH model, employing the fourth-order Runge–Kutta method (ode45) for numerical integration, with a maximum step size of 0.1 ms and a simulation duration of 500 ms. The externally applied input current *I*_ext_ was used as a unified depolarizing stimulation condition, upon which magnetic stimulation inputs with different parameters were superimposed.

In the revised simulations, the Control, 1 Hz-50 mT, 1 Hz-75 mT, 10 Hz-75 mT, and 20 Hz-75 mT conditions were included to evaluate whether the modified HH model could reproduce both the weak responses under low-frequency stimulation and the excitability-enhancing effects under high-frequency stimulation. The same model parameters, input current calibration, and numerical integration procedures were used across all simulated conditions. Membrane potential traces and AP thresholds were extracted and compared among different stimulation conditions. Simultaneously, the time-course changes in the sodium channel activation variable *m*, the sodium channel inactivation variable *h*, and the potassium channel activation gating variable *n* were further analyzed to compare the variation characteristics of gating kinetics in the two types of neurons before and after magnetic stimulation.

By calculating the steady-state values at different membrane potentials, the steady-state curves of the sodium and potassium channels were plotted. Specifically, the relative conductance (*G*/*G*_max_) on the ordinate represents the steady-state activation level of the channel at a given membrane potential, whereas the relative current (*I*/*I*_max_) represents the available fraction of channels in the non-inactivated state. Subsequently, the steady-state activation and inactivation curves were fitted using the Boltzmann equation:

(17) $$f(V)=1/\left\{1+\exp\left[\left(V_{m}-V_{1/2}\right)/k\right]\right\}$$

where *f*(*V*) is the relative conductance or relative current fraction at the corresponding membrane potential *V*; *V*_1/2_ is the half-activation (or half-inactivation) voltage, defined as the membrane potential at which the channel activation or inactivation reaches 50% of its maximum value, thereby reflecting the voltage threshold required for channel gating; and *k* is the slope factor, reflecting the steepness of the curve and the sensitivity of the channel gating process to voltage changes. By comparing the alterations in *V*_1/2_ and *k* before and after magnetic stimulation, the regulatory effects of magnetic stimulation on the voltage-dependent characteristics of ion channels were evaluated.

### 2.5. Statistical Analysis

Data analysis was performed using GraphPad Prism 10.1.2 statistical software. All statistical tests were two-tailed, with the significance level set at *p* < 0.05. To evaluate the effects of magnetic stimulation with different parameters on neuronal excitability indicators relative to the control group, a one-way analysis of variance (ANOVA) followed by Dunnett’s post hoc test was first employed to compare the differences between each stimulation frequency condition and the control group. To further examine the differences in effects between the 50 mT and 75 mT intensities under the same frequency conditions, as well as the differences among the 1 Hz, 10 Hz, and 20 Hz frequencies under the same intensity conditions, a two-way ANOVA was utilized, with frequency (1 Hz, 10 Hz, 20 Hz) and stimulation intensity (50 mT, 75 mT) as independent variables. When the main effect of intensity or the interaction effect reached significance, Šidák’s post hoc multiple comparisons test was applied to analyze the differences in intensity effects under different frequencies. When the main effect of frequency was significant, Tukey’s HSD post hoc test was used to compare the differences among various frequencies. Furthermore, to examine whether the modulation of neuronal excitability by magnetic stimulation with identical parameters differed between glutamatergic and GABAergic neurons, an additional two-way ANOVA was conducted, with stimulation parameters and cell type as independent variables. Upon significant effects, Šidák’s post hoc test was employed to analyze the differences in effects between different cell types under the same frequency condition. All data are presented as mean ± standard error of the mean (SEM). In addition to F statistics and *p* values, effect sizes were calculated as partial eta squared ($\eta_{p}^{2}$) for all ANOVA analyses.

## 3. Results

### 3.1. The Effects of Magnetic Stimulation on the Excitability of Neurons in Isolated Brain Slices

**Figure 3:**
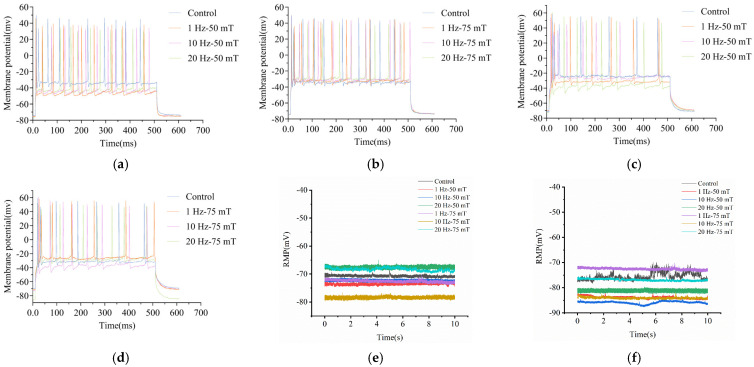
Diagram of AP firing frequency and RMP. (**a**–**d**) show membrane potential of glutamatergic and GABAergic neurons under 50 mT and 75 mT magnetic stimulation, respectively; panels (**e**,**f**) separately display resting membrane potential for glutamatergic and GABAergic neurons. (**a**) glutamatergic neurons under stimulation of 50 mT intensity; (**b**) glutamatergic neurons under 75 mT intensity; (**c**) GABAergic neurons under 50 mT intensity; (**d**) GABAergic neurons under 75 mT intensity. (**e**) glutamatergic neurons; (**f**) GABAergic neurons.

In this experiment, a 500-ms long-duration depolarizing current stimulation was applied to evoke APs, and the AP firing of the two types of neurons was recorded. Schematic diagrams of the long-duration evoked APs and RMPs for the two types of neurons under magnetic stimulation with different parameters are shown in Figure 3.

**Figure 4:**
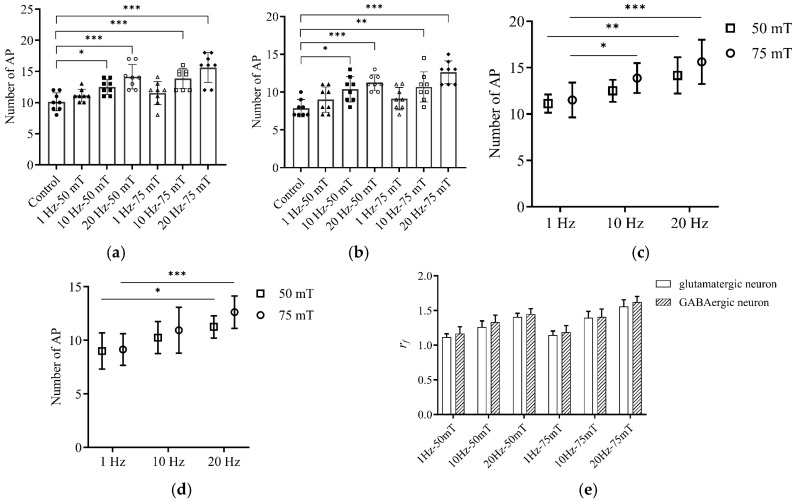
Number of APs. (**a**) Glutamatergic neurons. (**b**) GABAergic neurons. (**c**) Effects of frequency and intensity on the AP number of glutamatergic neurons. (**d**) Effects of frequency and intensity on the AP number of GABAergic neurons. (**e**) Ratio of AP numbers before and after stimulation for both neuron types (* *p* < 0.05, ** *p* < 0.01, *** *p* < 0.001; *n* = 8).

The effects of magnetic stimulation with different parameters on the number of APs are shown in Figure 4. Overall, high-frequency stimulation significantly increased the AP firing in both types of neurons. For glutamatergic neurons, compared to the Control group (10.12 ± 0.52), the number of APs significantly increased under all 10 Hz and 20 Hz stimulation conditions, peaking at 15.62 ± 0.84 in the 20 Hz-75 mT group (all *p* < 0.05 or *p* < 0.001; Figure 4a). A similar significant enhancement in firing was observed in GABAergic neurons under 10 Hz and 20 Hz conditions compared to the control (all *p* < 0.05 or *p* < 0.001; Figure 4b).

Two-way ANOVA revealed a significant main effect of stimulation frequency on AP counts for both glutamatergic (*F*(2, 42) = 16.98, *p* < 0.001, $\eta_{p}^{2}=0.45$) and GABAergic neurons (*F*(2, 42) = 13.00, *p* < 0.001, $\eta_{p}^{2}=0.38$), demonstrating a stepwise increase in spikes as frequency increased. Although the main effect of magnetic field intensity was also significant in glutamatergic neurons (*F*(1, 42) = 4.60, *p* = 0.038, $\eta_{p}^{2}=0.10$), post hoc analysis showed no significant differences between the 50 mT and 75 mT groups at the same frequency (Figure 4c,d).

To further evaluate these responses, we analyzed the ratio of post- to pre-stimulation AP counts (*r_f_*). The r*_f_* values increased in a frequency-dependent manner, reaching up to 1.56 ± 0.09 (a 56.0% increase) in glutamatergic neurons and 1.62 ± 0.08 (a 62.1% increase) in GABAergic neurons in the 20 Hz-75 mT group. Under identical stimulation parameters, no significant difference in r_f_ was found between the two neuron types (*F*(1, 84) = 0.88, *p* = 0.3509, $\eta_{p}^{2}=0.32$; Figure 4e).

Collectively, these results indicate that magnetic stimulation immediately enhances the firing activity of both DG neuron types. Within the tested parameter range, this immediate enhancement of excitability is primarily driven by stimulation frequency, whereas the further promotion of this effect by intensity changes is relatively limited.

### 3.2. The Effects of Magnetic Stimulation on the RMP of Neurons in Isolated Brain Slices

**Figure 5:**
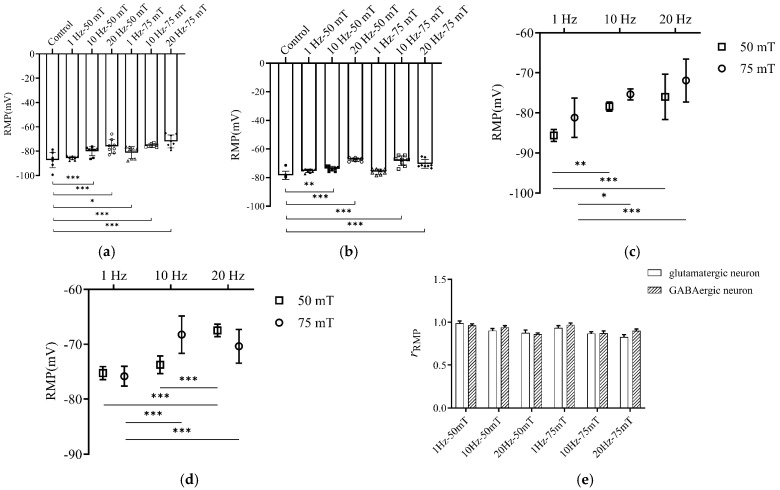
RMP. (**a**) Glutamatergic neurons. (**b**) GABAergic neurons. (**c**) Effects of frequency and intensity on the RMP of glutamatergic neurons. (**d**) Effects of frequency and intensity on the RMP of GABAergic neurons. (**e**) Ratio of RMP before and after stimulation in both neuron types. (* *p* < 0.05, ** *p* < 0.01, *** *p* < 0.001; *n* = 8).

The effects of magnetic stimulation with different parameters on the RMP of both neuron types are illustrated in Figure 5. Overall, high-frequency magnetic stimulation significantly depolarized the RMP in both types of neurons. For glutamatergic neurons, compared to the Control group, the RMP was significantly depolarized in all 10 Hz and 20 Hz groups, as well as in the 1 Hz-75 mT group (all *p* < 0.05 or *p* < 0.001; Figure 5a). Similarly, GABAergic neurons exhibited significantly increased RMPs under 10 Hz and 20 Hz conditions (all *p* < 0.01 or *p* < 0.001; Figure 5b).

Two-way ANOVA indicated a significant main effect of stimulation frequency on RMP for both glutamatergic (*F*(2, 42) = 24.98, *p* < 0.001, $\eta_{p}^{2}=0.54$) and GABAergic neurons (*F*(2, 42) = 37.41, *p* < 0.001, $\eta_{p}^{2}=0.64$). Although the main effect of magnetic field intensity was also significant for glutamatergic neurons (*F*(1, 42) = 11.90, *p* = 0.0013, $\eta_{p}^{2}=0.22$), the overall depolarization trend was predominantly driven by frequency (Figure 5c). In contrast, GABAergic neurons showed no significant main effect of intensity (*F*(1, 42) = 1.096, *p* = 0.3011, $\eta_{p}^{2}=0.03$; Figure 5d).

Analysis of the post- to pre-stimulation RMP ratio (*r*_RMP_) revealed no significant difference between the two neuron types under identical stimulation parameters (*F*(1, 84) = 1.934, *p* = 0.1680, $\eta_{p}^{2}=0.33$; Figure 5e). These findings suggest that magnetic stimulation immediately elevates the RMP of both neuron types, with high-frequency stimulation exerting a more pronounced effect, thereby enhancing neuronal excitability through increased membrane depolarization.

### 3.3. The Effects of Magnetic Stimulation on Individual AP Parameters in Neurons from Isolated Brain Slices

**Figure 6:**
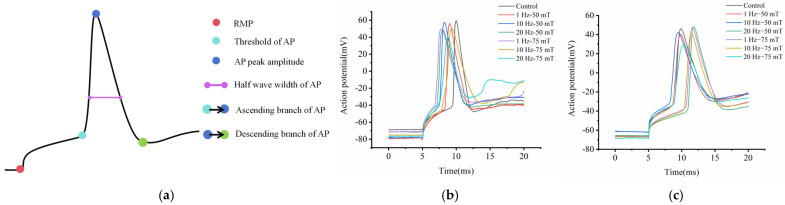
Diagram of a single AP. (**a**) AP-related parameters; (**b**) recorded waveforms of glutamatergic neurons; (**c**) recorded waveforms of GABAergic neurons.

While AP counts and RMP reflect the macroscopic modulatory effects of magnetic stimulation on overall neuronal excitability, the waveform parameters of individual APs are directly linked to the kinetics of voltage-gated ion channels, serving as critical indicators for elucidating the underlying mechanisms. To further explore whether magnetic stimulation regulates neuronal excitability by influencing processes such as AP initiation and repolarization, we systematically evaluated the impact of varying stimulation parameters on the AP characteristics of glutamatergic and GABAergic neurons. A schematic definition of these individual AP parameters is provided in Figure 6a, with representative AP traces before and after stimulation shown for glutamatergic (Figure 6b) and GABAergic neurons (Figure 6c).

#### 3.3.1. The Effect of Magnetic Stimulation on the AP Threshold

**Figure 7:**
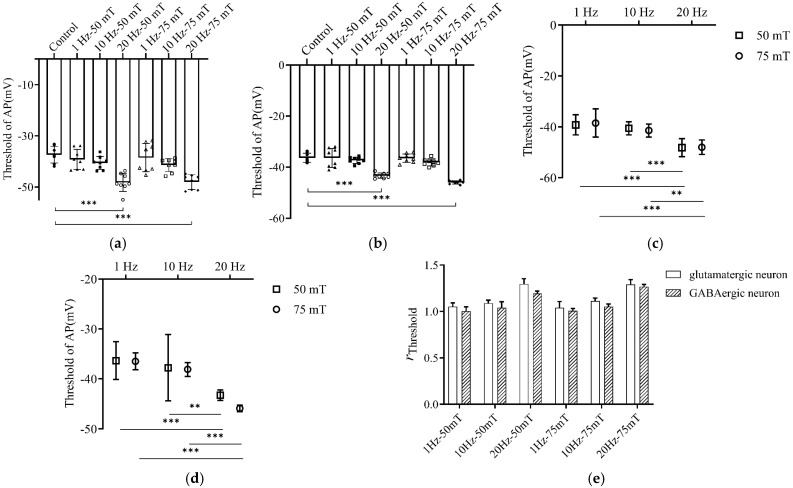
Analysis of AP threshold data. (**a**) Glutamatergic neurons. (**b**) GABAergic neurons. (**c**) Effects of frequency and intensity on the AP threshold of glutamatergic neurons. (**d**) Effects of frequency and intensity on the AP threshold of GABAergic neurons. (**e**) Ratio of AP thresholds before and after stimulation for both neuron types (** *p* < 0.01, *** *p* < 0.001; *n* = 8).

The impact of different magnetic stimulation parameters on the AP threshold of both neuron types is illustrated in Figure 7. Overall, 20 Hz stimulation significantly reduced the AP threshold compared to the control group in both glutamatergic and GABAergic neurons (all *p* < 0.001; Figure 7a,b).

Two-way ANOVA confirmed a significant main effect of frequency for both cell types (*F*(2, 42) = 27.89, $\eta_{p}^{2}=0.57$ for glutamatergic and 28.01 for GABAergic, $\eta_{p}^{2}=0.57$, both *p* < 0.001), whereas the main effect of intensity remained non-significant (*p* > 0.05; Figure 7c,d). Post hoc analysis further revealed that the threshold at 20 Hz was significantly lower than at 1 Hz and 10 Hz across all intensity levels.

Additionally, analysis of the post- to pre-stimulation threshold ratio (r_Threshold_) showed no significant difference between glutamatergic and GABAergic neurons under identical parameters (Figure 7e). These findings demonstrate that the modulation of the AP threshold is primarily frequency-dependent, with 20 Hz being the most effective parameter, while variations in field intensity have a negligible influence.

#### 3.3.2. The Effect of Magnetic Stimulation on AP Peaks

AP peak was defined as the maximum membrane potential (mV) reached during the action potential.

**Figure 8:**
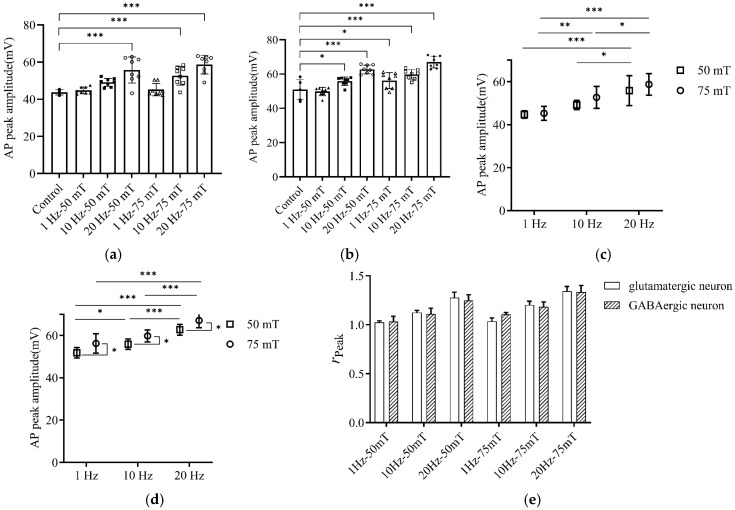
AP peak data analysis. (**a**) Glutamatergic neurons. (**b**) GABAergic neurons. (**c**) Effects of frequency and intensity on the peak of glutamatergic APs. (**d**) Effects of frequency and intensity on the peak of GABAergic APs. (**e**) Pre- and post-stimulation ratios of peak AP amplitudes in both neuron types (* *p* < 0.05, ** *p* < 0.01, *** *p* < 0.001; *n* = 8).

The impact of different magnetic stimulation parameters on the AP peak of both neuron types is illustrated in Figure 8. Overall, high-frequency stimulation significantly increased the AP peak in both types of neurons. For glutamatergic neurons, compared to the control group, the AP peak was significantly elevated in the 20 Hz-50 mT, 10 Hz-75 mT, and 20 Hz-75 mT groups (all *p* < 0.001; Figure 8a). Similarly, GABAergic neurons exhibited significantly increased AP peaks in all 10 Hz and 20 Hz groups, as well as the 1 Hz-75 mT group (all *p* < 0.05 or *p* < 0.001; Figure 8b).

Two-way ANOVA revealed a highly significant main effect of stimulation frequency on the AP peak for both glutamatergic (*F*(2, 42) = 30.40, *p* < 0.001, $\eta_{p}^{2}=0.59$) and GABAergic neurons (*F*(2, 42) = 49.25, *p* < 0.001, $\eta_{p}^{2}=0.70$), demonstrating a frequency-dependent increase. Notably, the main effect of magnetic field intensity was only significant in GABAergic neurons (*F*(1, 42) = 21.66, *p* < 0.001, $\eta_{p}^{2}=0.34$), where post hoc analysis showed that the peaks in the 75 mT groups were significantly higher than those in the corresponding 50 mT groups across all frequencies (all *p* < 0.05; Figure 8d). In contrast, the main effect of intensity was not significant in glutamatergic neurons (*p* = 0.0819; Figure 8c).

Analysis of the post- to pre-stimulation AP peak ratio (r_Peak_) showed no significant difference between the two neuron types under identical stimulation parameters (*F*(1, 84) = 0.002, *p* = 0.9644, $\eta_{p}^{2}=0.44$; Figure 8e). These results indicate that while the enhancement of the AP peak is primarily driven by stimulation frequency, GABAergic neurons are more sensitive to changes in stimulation intensity than glutamatergic neurons.

#### 3.3.3. The Effect of Magnetic Stimulation on the AP Half-Width

**Figure 9:**
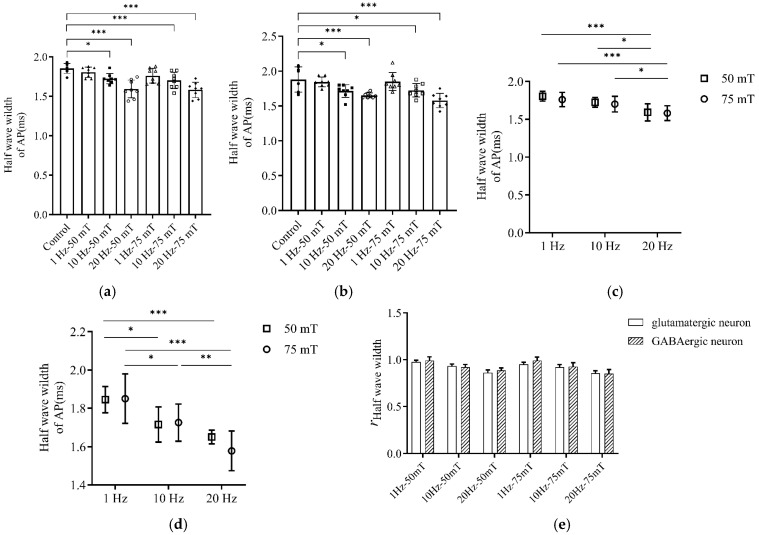
Analysis of AP half width data. (**a**) glutamatergic neurons; (**b**) GABAergic neurons; (**c**) Effects of frequency and intensity on the half-width of glutamatergic APs; (**d**) Effects of frequency and intensity on the half-width of GABAergic APs; (**e**) Ratio of AP half-widths before and after stimulation for both neuron types (* *p* < 0.05, ** *p* < 0.01, *** *p* < 0.001; *n* = 8).

The effects of different magnetic stimulation parameters on the AP half-width of both neuron types are presented in Figure 9. Overall, high-frequency stimulation significantly reduced the AP half-width. Compared to the control group, both glutamatergic and GABAergic neurons exhibited significantly shortened half-widths under all 10 Hz and 20 Hz stimulation conditions (all *p* < 0.05 or *p* < 0.001; Figure 9a,b).

Two-way ANOVA revealed a significant main effect of stimulation frequency on AP half-width for both glutamatergic (*F*(2, 42) = 18.96, *p* < 0.001, $\eta_{p}^{2}=0.47$) and GABAergic neurons (*F*(2, 42) = 25.76, *p* < 0.001, $\eta_{p}^{2}=0.55$). Post hoc analysis further confirmed that 20 Hz stimulation was the most effective parameter for reducing half-width across all intensity levels. In contrast, the main effect of magnetic field intensity remained non-significant for both neuron types (both *p* > 0.05; Figure 9c,d).

Analysis of the post- to pre-stimulation half-width ratio (r_Half-width_) showed no significant difference between the two neuron types under identical stimulation parameters (*F*(1, 84) = 0.0216, *p* = 0.0884, $\eta_{p}^{2}=0.24$; Figure 9e). These findings indicate that the modulation of AP half-width is primarily frequency-dependent. By accelerating the AP time course, magnetic stimulation enhances the instantaneous firing capacity and excitability of both neuron types.

#### 3.3.4. The Effect of Magnetic Stimulation on the Maximum Rise Slope of the AP

**Figure 10:**
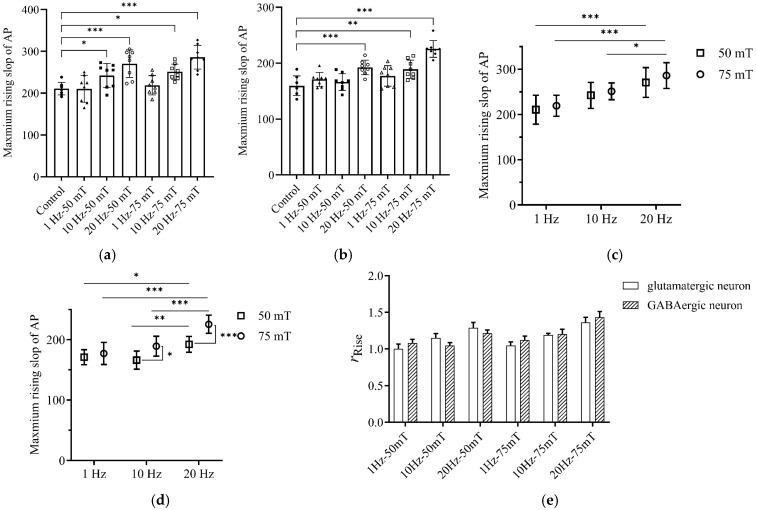
Data analysis of AP ascending branch maximum slope. (**a**) Glutamatergic neurons. (**b**) GABAergic neurons. (**c**) Effects of frequency and intensity on the maximum slope of the ascending branch of glutamatergic APs. (**d**) Effects of frequency and intensity on the maximum slope of the ascending branch of GABAergic APs. (**e**) Ratio of the maximum slope of the ascending branch of APs before and after stimulation for both neuron types (* *p* < 0.05, ** *p* < 0.01, *** *p* < 0.001; *n* = 8).

The effects of magnetic stimulation on the maximum rising slope of APs are presented in Figure 10. High-frequency stimulation significantly increased the maximum rising slope in both neuron types. For glutamatergic neurons, significant increases were observed in all 10 Hz and 20 Hz stimulation groups compared to the control group (all *p* < 0.05 or *p* < 0.001; Figure 10a). GABAergic neurons showed a similar trend, with significantly higher rising slopes under 20 Hz-50 mT and 10–20 Hz-75 mT conditions (all *p* < 0.01 or *p* < 0.001; Figure 10b).

Two-way ANOVA revealed a significant main effect of stimulation frequency on the maximum rising slope for both glutamatergic (*F*(2, 42) = 20.9, *p* < 0.001, $\eta_{p}^{2}=0.50$) and GABAergic neurons (*F*(2, 42) = 25.53, *p* < 0.001, $\eta_{p}^{2}=0.55$). Notably, a significant main effect of magnetic field intensity was observed only in GABAergic neurons (*F*(1, 42) = 22.63, *p* < 0.001, $\eta_{p}^{2}=0.35$), where the 10 Hz and 20 Hz groups at 75 mT exhibited significantly higher slopes than their corresponding 50 mT groups (Figure 10d). For glutamatergic neurons, the intensity effect was non-significant (*p* = 0.177; Figure 10c).

Analysis of the post- to pre-stimulation rising slope ratio (r_Rise_) showed no significant difference between the two cell types under identical parameters (Figure 10e). These results indicate that magnetic stimulation accelerates the depolarization phase of APs in both neuron types, primarily driven by frequency, while GABAergic neurons demonstrate higher sensitivity to stimulation intensity.

#### 3.3.5. Effects on AP Maximum Falling Slope

**Figure 11:**
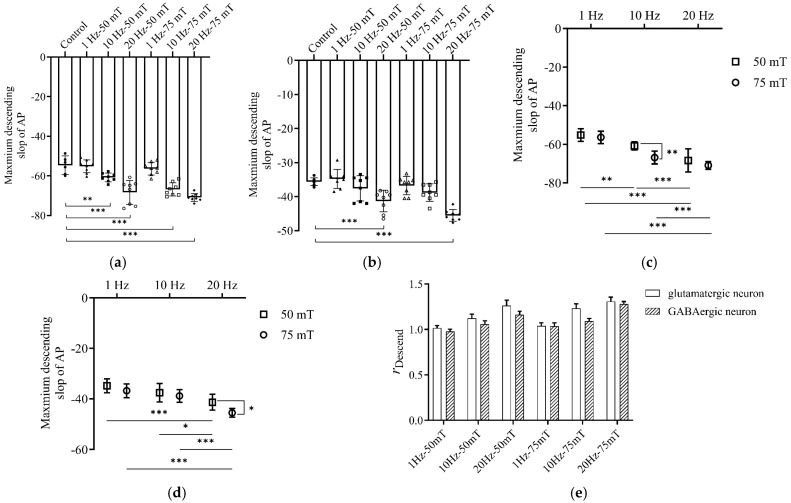
Data analysis of maximum slope of AP descending branch. (**a**) Glutamatergic neurons. (**b**) GABAergic neurons. (**c**) Effects of frequency and intensity on the maximum slope of the descending branch of glutamatergic AP. (**d**) Effects of frequency and intensity on the maximum slope of the descending branch of GABAergic AP. (**e**) Ratio of the maximum slope of the descending branch of AP before and after stimulation for both neuron types (* *p* < 0.05, ** *p* < 0.01, *** *p* < 0.001; *n* = 8).

The effects of different magnetic stimulation parameters on the maximum falling slope of APs are illustrated in Figure 11. Overall, high-frequency magnetic stimulation significantly reduced the absolute maximum falling slope of APs, indicating slowed membrane repolarization during the downstroke phase. in both neuron types. Compared to the control group, the maximum falling slope in glutamatergic neurons was significantly reduced under all 10 Hz and 20 Hz conditions (all *p* < 0.01 or *p* < 0.001; Figure 11a). GABAergic neurons exhibited a similar significant reduction under 20 Hz conditions (all *p* < 0.001; Figure 11b).

Two-way ANOVA revealed that both stimulation frequency and magnetic field intensity had significant main effects on the maximum falling slope across both cell types. The main effect of frequency was highly significant (glutamatergic: *F*(2, 42) = 60.34, $\eta_{p}^{2}=0.74$; GABAergic: *F*(2, 42) = 30.23, $\eta_{p}^{2}=0.59$, both *p* < 0.001). Furthermore, unlike some earlier kinetic parameters, the main effect of intensity was also significant for both glutamatergic (*F*(1, 42) = 9.851, *p* = 0.003, $\eta_{p}^{2}=0.19$) and GABAergic neurons (*F*(1, 42) = 9.08, *p* = 0.0044, $\eta_{p}^{2}=0.18$). Post hoc analysis indicated that stimulation at 75 mT further decreased the falling slope compared to 50 mT under high-frequency conditions (Figure 11c,d).

Analysis of the post- to pre-stimulation falling slope ratio (*r*_Downstroke_) revealed no significant difference between the two neuron types under identical stimulation parameters (Figure 11e). These results suggest that magnetic stimulation significantly alters the repolarization kinetics of APs in both neuron types, with both frequency and intensity playing crucial modulatory roles.

### 3.4. Simulation Results of Magnetic Stimulation on Neuronal Excitability

#### 3.4.1. Determination of I_ext_ and Model Validation

**Figure 12:**
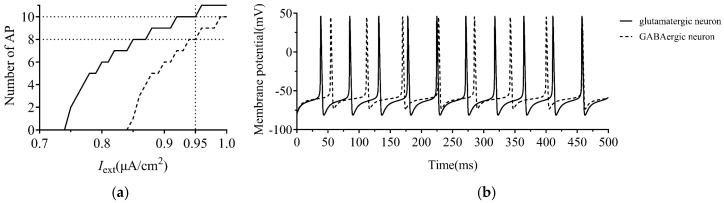
Firing frequencies of two types of neurons under different input current conditions. (**a**) Number of neuronal spikes; (**b**) neuronal membrane potential at *I*_ext_ = 0.95 μA/cm^2^.

During model validation, the effect of the external input current *I*_ext_ on the firing behavior of both neuron types was first evaluated. As shown in Figure 12a, the AP firing frequencies of both glutamatergic and GABAergic neurons gradually increased with higher *I*_ext_, indicating that the model reflects the modulation of neuronal excitability by input currents. Under identical *I*_ext_ conditions, glutamatergic neurons exhibited a slightly higher overall firing frequency than GABAergic neurons, reflecting differences in their baseline excitability. To maintain consistent control variables in the simulation analysis—mirroring the identical external current stimulation applied to both neuron types in the patch-clamp experiments—we uniformly set *I*_ext_ = 0.95 μA/cm^2^ for subsequent simulations. This specific value was chosen to align the model’s baseline output closely with the firing levels observed in the experimental control group.

Under this unified input current, both neuron types exhibited stable, periodic firing in the absence of a magnetic field (Figure 12b). Specifically, glutamatergic neurons displayed a higher baseline firing frequency than GABAergic neurons. Both cell types demonstrated clear AP waveforms and stable rhythms, with distinct differences in peak, potential change rate, and inter-spike intervals, all of which were consistent with the experimental recordings. Furthermore, the simulated AP peaks under control conditions (without magnetic stimulation) were approximately 43 mV for glutamatergic neurons and 48 mV for GABAergic neurons, closely matching the experimental measurements. These results demonstrate that under the selected parameter settings, the model successfully reproduces both the baseline firing behaviors and the AP amplitude characteristics of the two neuron types, thereby validating the rationality of the model.

#### 3.4.2. Simulation of AP Threshold and Spike Number Under Magnetic Stimulation

**Figure 13:**
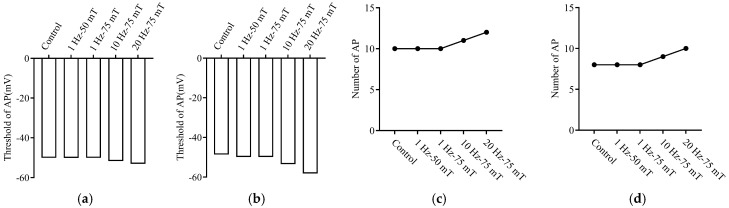
Simulation results of AP threshold and spike number under magnetic stimulation. (**a**) AP threshold of glutamatergic neurons; (**b**) AP threshold of GABAergic neurons; (**c**) number of APs in glutamatergic neurons; (**d**) number of APs in GABAergic neurons.

As illustrated in Figure 13, the modified HH model was further used to evaluate AP threshold and spike number under different magnetic stimulation conditions. Compared with the Control condition, the 1 Hz-50 mT and 1 Hz-75 mT groups showed only minimal changes in AP threshold and spike number in both glutamatergic and GABAergic neurons. This indicates that low-frequency stimulation produced weak modulatory effects in the simulation.

In contrast, the 10 Hz-75 mT and 20 Hz-75 mT conditions produced more evident changes in neuronal firing behavior. Specifically, high-frequency stimulation decreased the AP threshold and increased the number of AP spikes in both neuron types, with the effect being more pronounced under the 20 Hz-75 mT condition. These simulated trends are consistent with the experimental observations, in which high-frequency stimulation significantly enhanced neuronal excitability, whereas the low-frequency conditions produced relatively weak effects. Although minor numerical differences naturally exist between computational models and biological tissues, the modified HH model reproduced the parameter-dependent pattern of excitability modulation, thereby supporting its reliability for subsequent ion-channel mechanism analysis.

#### 3.4.3. Simulation of Ion-Channel Gating Variables Under Magnetic Stimulation

**Figure 14:**
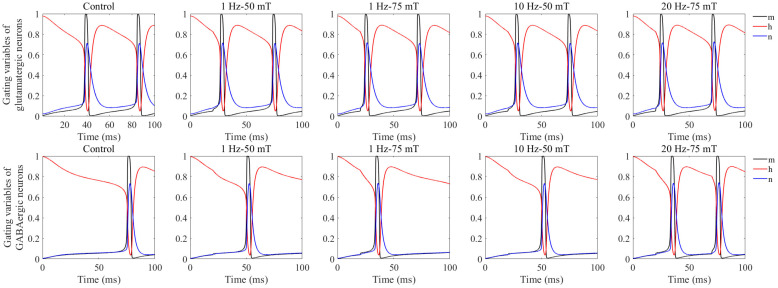
Simulation results of magnetic stimulation on ion channel gating variables.

To further elucidate the ion-channel mechanisms underlying excitability modulation, we compared the temporal dynamics of the sodium channel activation variable m, sodium channel inactivation variable h, and potassium channel activation variable *n* under different stimulation conditions (Figure 14).

Under the Control condition, the gating variables of both glutamatergic and GABAergic neurons exhibited regular periodic fluctuations corresponding to stable repetitive firing. Under the 1 Hz-50 mT and 1 Hz-75 mT conditions, the temporal profiles of m, h, and *n* remained very close to those of the Control condition, indicating that low-frequency stimulation had little effect on the dynamic gating processes of sodium and potassium channels.

By contrast, under high-frequency stimulation, especially in the 10 Hz-75 mT and 20 Hz-75 mT groups, the dynamic fluctuations of the gating variables became more frequent and more rapid. The sodium activation variable m showed more frequent rapid activation, the sodium inactivation variable h exhibited accelerated decline and recovery phases, and the potassium activation variable *n* displayed more pronounced periodic oscillations. These changes corresponded to the increased AP firing observed in the simulation results. Therefore, the gating-variable analysis suggests that the excitability-enhancing effect of magnetic stimulation is mainly associated with high-frequency stimulation-induced acceleration of sodium and potassium channel gating kinetics.

#### 3.4.4. Steady-State Ion-Channel Properties Under Magnetic Stimulation

**Figure 15:**
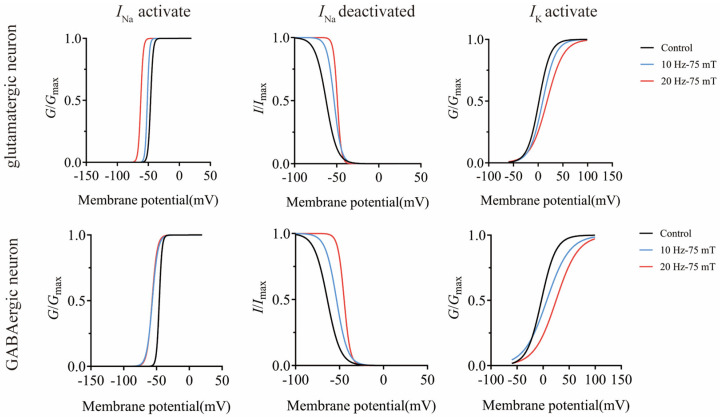
Simulation results of the effects of magnetic stimulation on neuronal ion channels. Simulations for the 1 Hz-50 mT and 1 Hz-75 mT conditions were also performed. Because the simulated potassium gating variable (*n*) almost completely overlapped with the Control condition, these traces are not shown for clarity.

To further evaluate the impact of magnetic stimulation on the voltage-dependent properties of ion channels, we extracted the steady-state activation and inactivation parameters for *I*_Na_ and *I*_K_ based on the simulation results (Figure 15, Table 3, Table 4 and Table 5).

The simulations showed that the 1 Hz-50 mT and 1 Hz-75 mT conditions produced almost no changes in the steady-state ion-channel parameters compared with the Control condition. In Table 3, Table 4 and Table 5, the *V*_1/2_ and slope factor *k* values of the low-frequency groups were nearly identical to those of the Control group, indicating that 1 Hz stimulation had a limited effect on the voltage-dependent gating properties of sodium and potassium channels. In Figure 15, the simulated potassium activation variable under the 1 Hz conditions also almost completely overlapped with the Control condition; therefore, these traces were not displayed separately for clarity.

In contrast, high-frequency stimulation induced clear changes in the steady-state properties of ion channels. For sodium channels, the activation curve shifted toward more negative membrane potentials, suggesting that sodium channels could be activated more readily during depolarization. Meanwhile, the sodium channel inactivation curve shifted toward more depolarized potentials, indicating that a larger fraction of sodium channels remained available in the non-inactivated state at a given membrane potential. For potassium channels, the activation curve shifted toward more depolarized potentials, suggesting a delayed activation process requiring higher voltage levels.

Although *V*_1/2_ exhibited the most consistent changes under high-frequency stimulation, the slope factor *k* also showed condition- and channel-dependent alterations. In particular, a marked increase in the *k* value of the sodium activation curve was observed in GABAergic neurons under the 10 Hz-75 mT condition, whereas the remaining *k* values exhibited relatively smaller changes. Overall, the predominant effect of magnetic stimulation was reflected in the shifts in *V*_1/2_ rather than uniform changes in *k*, indicating that magnetic stimulation mainly altered the voltage threshold required for channel gating. Collectively, these results suggest that low-frequency stimulation has little influence on channel gating properties, whereas high-frequency stimulation more effectively modifies the voltage-dependent activation and inactivation characteristics of sodium and potassium channels. This provides a possible channel-level explanation for the frequency-dependent enhancement of neuronal excitability observed in the experiments.

## 4. Discussion

Neuronal excitability, defined as the capacity to generate APs in response to stimuli, represents the fundamental dynamic transition between resting and depolarized states, directly governing neural information coding and transmission [33]. Aberrant excitability in the DG is closely linked to cognitive impairment, critically impacting hippocampal information processing and memory-related functions [34,35]. Consequently, modulating neuronal excitability in the DG has attracted considerable interest in neuromodulation research. Although our findings may help improve the understanding of cellular responses to magnetic stimulation, caution should be exercised when extrapolating these results to clinical rTMS because the induced electric fields used in this in vitro study were substantially lower than those typically associated with direct neuronal activation in human rTMS. This study elucidates the immediate modulatory characteristics of magnetic stimulation on glutamatergic and GABAergic neurons in the hippocampal DG region and explores the underlying mechanisms utilizing a modified Hodgkin–Huxley (HH) model.

Our findings demonstrate that magnetic stimulation immediately enhances the excitability of both neuron types, characterized by increased AP firing, depolarized RMP, and altered individual AP waveform parameters. Notably, this modulation is highly frequency-dependent. High-frequency stimulation (10–20 Hz) exerted a pronounced facilitatory effect, whereas the influence of intensity variations within the 50–75 mT range was relatively limited. This indicates that within the tested parameter spectrum, frequency serves as the primary driver for immediate excitability changes, acting as the core determinant. These macroscopic trends align well with previous studies in hippocampal slices and DG granule cells, which similarly reported that high-frequency magnetic stimulation is more effective at enhancing neuronal excitability [35].

Furthermore, our analysis revealed striking cell-type-specific divergent responses to magnetic stimulation. Glutamatergic neurons primarily exhibited a strict, frequency-dependent increase in macroscopic AP firing counts. In contrast, GABAergic neurons not only showed enhanced firing activity but also demonstrated significantly greater sensitivity and dynamic remodeling in intrinsic AP waveform parameters, such as the threshold, half-width, and rising/falling slopes. This suggests that the modulatory effects of magnetic stimulation are not uniformly distributed but are profoundly shaped by the distinct intrinsic membrane properties, ion channel compositions, and firing dynamics of different cell populations. This perspective is corroborated by recent literature indicating that repetitive brain stimulation can induce differential and asymmetric excitability changes between glutamatergic and GABAergic populations [36].

From the perspective of AP waveform modifications, the observed decrease in threshold, increase in peak, and accelerated kinetics (altered half-width and steepened maximum rising/falling slopes) suggest that magnetic stimulation not only promotes firing probability but also profoundly reshapes the entire process of AP initiation and repolarization [37]. The lowering of the AP threshold—the critical tipping point for “all-or-none” firing [38]—indicates an increased susceptibility to activation, which is fundamentally governed by the kinetics of voltage-gated sodium channels [39,40]. The induced electric field or eddy currents generated by magnetic stimulation may alter the local membrane potential distribution, thereby modifying the conformational dynamics of sodium channels. This modulation allows them to activate at more negative membrane potentials or recover more rapidly from inactivation, consequently lowering the AP threshold.

This mechanistic hypothesis is further supported by the modified HH model analysis. Importantly, after adding the 1 Hz-50 mT and 1 Hz-75 mT simulation conditions, the model reproduced not only the excitability-enhancing effects under high-frequency stimulation but also the weak responses under low-frequency stimulation. Under the 1 Hz conditions, AP firing, gating-variable dynamics, and steady-state ion-channel parameters remained close to those of the Control condition. This result is consistent with the relatively limited electrophysiological changes observed under low-frequency stimulation and provides a more stringent validation of the model. In contrast, under high-frequency stimulation, the transition processes of the sodium activation gate, sodium inactivation gate, and potassium activation gate were accelerated, and these changes were accompanied by increased AP firing. Since the coordinated activation, inactivation, and recovery of sodium channels, together with potassium channel activation, constitute the core biophysical basis of AP generation and repolarization [41,42], these simulation results provide a possible explanation for the experimentally observed enhancement of neuronal excitability. The steady-state analysis further showed that high-frequency magnetic stimulation altered the voltage-dependent properties of ion channels. Sodium channel activation shifted toward more negative potentials, which may facilitate AP initiation, whereas sodium channel inactivation shifted toward more depolarized potentials, allowing a larger fraction of sodium channels to remain available at a given membrane potential. Potassium channel activation shifted toward more depolarized potentials, suggesting a delayed activation process during membrane depolarization. Together, these findings indicate that magnetic stimulation does not uniformly enhance neuronal excitability under all parameter conditions. Instead, its effect is strongly frequency-dependent, with high-frequency stimulation producing more pronounced modulation of ion-channel kinetics and neuronal firing behavior.

The long-term therapeutic effects of magnetic stimulation likely originate from its immediate modulation of neuronal excitability. Consistent with our current findings, previous studies by our group and others have demonstrated that acute high-frequency rTMS enhances the excitability and Na^+^/K^+^ channel activity of DG granule cells, thereby facilitating electrical signaling and boosting network function [43]. Importantly, beyond alterations in intrinsic AP generation, these immediate effects are likely mediated by a multifaceted synergy of synaptic and intracellular mechanisms. For instance, magnetic stimulation can modulate voltage-gated calcium channels to alter presynaptic neurotransmitter release probability [44], and affect ligand-gated ion channels, such as NMDA receptors, to tune postsynaptic excitatory inputs [45]. Furthermore, the induced membrane potential fluctuations may trigger intracellular signaling cascades, utilizing second messengers to regulate downstream protein kinases and effector molecules. These mechanisms are beyond the scope of the present study and should be considered as potential explanations to be examined in future studies. Thus, the immediate modulation of neuronal excitability is a highly integrated process encompassing intrinsic channel kinetics, presynaptic transmitter release, postsynaptic receptor regulation, and intracellular signaling pathways.

Several limitations of the current study should be acknowledged. Computationally, although the modified HH model successfully reproduced the experimentally observed trends in firing frequency and AP waveforms, it remains a highly simplified single-compartment model. This simplification inherently neglects more complex biophysical processes, such as dendritic morphological effects and synaptic current integration [46], which warrant further investigation in future multi-compartmental network models.

At the human level, intracranial single-unit recordings confirmed that prefrontal TMS modulates putative pyramidal cells and interneurons with divergent directional effects across cell classes [47]. In awake mice, prefrontal rTMS drives specific plasticity in intratelencephalic but not pyramidal-tract neurons to drive antidepressant effects [48], with circuit-level fronto-insular mechanisms further detailed under accelerated stimulation [49].

A major point of convergence is that magnetic fields selectively interact with specific neuronal subclasses based on their biophysical identities [47]. A point of divergence is that while those studies focus on long-term network plasticity and behaviors in awake animals [48,49], our study isolates the immediate, sub-millisecond acceleration of ion-channel gating kinetics in acute slices. Together, these cross-scale insights complement our baseline cellular findings.

Several limitations of the present study should be acknowledged. First, neuronal identity was determined based on cell-type-specific viral labeling without post hoc immunohistochemical validation. Although the viral vectors used have been widely validated in previous studies, dentate gyrus GABAergic interneurons comprise heterogeneous subtypes with distinct electrophysiological properties. In addition, all experiments were performed in acute hippocampal slices obtained from adult male mice. Therefore, the present findings should be interpreted within the context of an in vitro preparation and may not fully reflect sex-dependent or network-level responses in vivo.

Second, the present study focused on the immediate effects of magnetic stimulation on the intrinsic excitability of individual neurons. Synaptic transmission, voltage-clamp characterization of ion-channel kinetics, and behavioral outcomes were not investigated. Consequently, the observed changes should be interpreted as alterations in intrinsic membrane excitability rather than direct evidence of changes in synaptic function, neural circuit activity, or behavioral performance.

Third, the modified Hodgkin–Huxley model adopted in this study was based on the computational framework proposed by Yang et al. and should be regarded as a phenomenological model. The magnetic-field-dependent current terms represent an effective mathematical description of the experimental observations rather than a rigorous first-principles electromagnetic mechanism. In addition, because granule-cell-specific Hodgkin–Huxley parameters remain limited, the glutamatergic neuron model was parameterized using electrophysiological properties of CA1 pyramidal neurons. Therefore, the simulated changes in ion-channel kinetics should be interpreted as qualitative computational predictions rather than precise quantitative estimates.

Finally, the stimulation conditions investigated here were limited to frequencies between 1 and 20 Hz, and the induced electric field represented subthreshold magnetic stimulation in an in vitro preparation. Furthermore, although coil heating was minimal and brain slices were continuously perfused, bath temperature was not monitored directly during stimulation. Future studies incorporating broader stimulation parameters, real-time thermal monitoring, more comprehensive electrophysiological validation, and in vivo functional assessments will further improve the translational significance of these findings.

## 5. Conclusions

In conclusion, the present study demonstrates that magnetic stimulation immediately enhances the excitability of both glutamatergic and GABAergic neurons in the hippocampal DG region. This enhancement is primarily characterized by increased AP firing, depolarized RMP, and altered AP waveform kinetics. High-frequency stimulation exhibits a more pronounced facilitatory effect, whereas the influence of magnetic field intensity within the 50–75 mT range remains relatively limited. Notably, the two cell types exhibit divergent response profiles: glutamatergic neurons primarily show a strict frequency-dependent enhancement in macroscopic firing activity, whereas GABAergic neurons display greater parameter sensitivity in their RMPs and intrinsic AP waveform characteristics. Furthermore, computational simulations showed that the 1 Hz-50 mT and 1 Hz-75 mT conditions produced little change compared with the Control condition, whereas high-frequency stimulation induced more evident alterations in AP firing, gating-variable dynamics, and steady-state ion-channel properties. These findings further support the frequency-dependent nature of magnetic stimulation effects. At the mechanistic level, the immediate enhancement of neuronal excitability may be associated with accelerated gating kinetics and altered voltage-dependent properties of voltage-gated sodium and potassium channels under high-frequency stimulation.

## Figures and Tables

**Table 1:** Solution formulation.

Solution	Composition (m mol/L)	pH
ACSF	CaCl_2_ 3, NaCl 125, KCl 4, MgCl_2_ 3, NaH_2_PO_4_ 1.635, NaHCO_3_ 28, HEPES 5, glucose 11	7.4 (NaOH)
Internal solution of AP	K-gluconate 126, NaCl 16, MgCl_2_ 3, CaCl_2_ 1.5, EGTA 12, HEPES 11, Na-ATP 4, Na-GTP 0.4	7.4 (KOH)
Cutting solution	MgCl_2_ 7, CaCl_2_ 2, KCl 3, NaHCO_3_ 28, NaH_2_PO_4_ 1.655, glucose 13, sucrose 220	7.4 (NaOH)

**Table 3:** Kinetic parameters of the simulated *I*_Na_ activation curve.

Group	Glutamatergic Neuron		GABAergic Neuron	
	Half Activation Voltage *V*_1/2_ (mV)	Slope Factor *k*	Half Activation Voltage *V*_1/2_ (mV)	Slope Factor *k*
Control	−46.30	2.05	−45.76	2.09
1 Hz-50 mT	−46.30	2.05	−45.76	2.09
1 Hz-75 mT	−46.30	2.05	−45.76	2.09
10 Hz-75 mT	−51.57	1.73	−56.37	4.29
20 Hz-75 mT	−62.17	2.05	−56.74	3.85

**Table 4:** Kinetic parameters of the simulated *I*_Na_ inactivation curve.

Group	Glutamatergic Neuron		GABAergic Neuron	
	Half Inactivation Voltage *V*_1/2_ (mV)	Slope Factor *k*	Half Inactivation Voltage *V*_1/2_ (mV)	Slope Factor *k*
Control	−62.54	6.49	−63.87	7.44
1 Hz-50 mT	−62.54	6.49	−63.87	7.44
1 Hz-75 mT	−62.54	6.49	−63.87	7.44
10 Hz-75 mT	−53.11	4.26	−53.43	6.33
20 Hz-75 mT	−49.18	2.33	−44.78	3.51

**Table 5:** Kinetic parameters of the simulated *I*_K_ activation curve.

Group	Glutamatergic Neuron		GABAergic Neuron	
	Half Activation Voltage *V*_1/2_ (mV)	Slope Factor *k*	Half Activation Voltage *V*_1/2_ (mV)	Slope Factor *k*
Control	1.79	11.61	−3.23	13.9
1 Hz-50 mT	1.79	11.61	−3.23	13.9
1 Hz-75 mT	1.79	11.61	−3.23	13.9
10 Hz-75 mT	9.40	13.15	7.10	21.8
20 Hz-75 mT	18.15	16.7	24.96	21.06

## Data Availability

The data presented in this study are not publicly available due to institutional data management policies but are available from the corresponding author upon reasonable request.

## Abbreviations

The following abbreviations are used in this manuscript:

DG	Dentate gyrus
HH	Hodgkin–Huxley
RMP	Resting Membrane Potential
AP	Action Potential
rTMS	Repetitive transcranial magnetic stimulation
ACSF	Artificial Cerebrospinal Fluid
